# Comparison of two methods of bed-to/from-wheelchair transfer in patients with hemiparetic stroke

**DOI:** 10.20407/fmj.2019-016

**Published:** 2020-02-11

**Authors:** Soichiro Koyama, Shigeo Tanabe, Eiichi Saitoh, Yohei Otaka, Hirofumi Ohta, Tsuyoshi Tatemoto, Nobuhiro Kumazawa, Ai Katoh, Yuki Sugiyama, Kei Kiyono, Yoshikiyo Kanada

**Affiliations:** 1 Faculty of Rehabilitation, School of Health Sciences, Fujita Health University, Toyoake, Aichi, Japan; 2 Department of Rehabilitation Medicine I, School of Medicine, Fujita Health University, Toyoake, Aichi, Japan; 3 Department of Rehabilitation, Fujita Health University Hospital, Toyoake, Aichi, Japan

**Keywords:** Stroke, Wheelchair users, Transfer method, Lateral, Hemiparetic

## Abstract

**Objectives:**

The ability to transfer between surfaces is essential for wheelchair users’ independence. We hypothesized that transfer of hemiparetic stroke patients would be improved by using surfaces at the same height with no gap or obstacle between them.

**Methods:**

A cross-sectional study was conducted to compare the difficulty of two transfer methods as a pilot study. Thirteen hemiparetic stroke patients were transferred from a platform table to a chair (wheelchair or flat chair) and from the chair to the table using the regular and lateral transfer methods. Functional Independence Measure (FIM) transfer score in both transfer methods and Stroke Impairment Assessment Set (SIAS) score were measured.

**Results:**

The FIM transfer score significantly increased in the lateral transfer condition compared with the regular transfer condition, indicating that the former method reduced the transfer difficulty, regardless of the SIAS scores.

**Conclusions:**

The transfer difficulty of patients with hemiparetic stroke decreases when using the lateral transfer method. The lateral transfer method is easy, potentially helping prevent care-related injuries among caregivers.

## Introduction

Mobility is a fundamental function for the enrichment of well-being and quality of life, and the ability to transfer from one surface to another is essential for independence among wheelchair users. Despite transfers being a frequent daily activity, they are ranked as difficult among wheelchair-related activities.^1,2^

A previous study demonstrated that the difficulty of performing transfers is affected by the difference in height, width of the gap, and presence of an obstacle (armrest) between transfer surfaces.^3^ Specifically, in cases where the person is able to independently perform a transfer to and from a wheelchair, gaps wider than 8.9 cm between transfer surfaces at the same height make transfers increasingly difficult, particularly in the presence of an obstacle.^3^ The presence of such difficulties between transfer surfaces requires greater recruitment of the biceps and anterior deltoid muscles compared with when a wheelchair is located close to a transfer surface.^4–6^

Moreover, transfer activity can cause upper limb pain and overuse-related injuries in wheelchair users,^7–9^ and is the most common activity leading to falls in patients with stroke.^10^ In a multivariate analysis performed by Lamb et al., transfer ability was related to the incidence of falls.^11^

Difficulties in performing transfers in wheelchair users can cause additional problems for the healthcare provider and/or family members who provide care for them. For persons who are not able to transfer by themselves, a caregiver is required to support transfers during daily activities. A previous study reported that work-related injuries to healthcare workers and caregivers during transfers are common.^12–18^ Providing assistance with transfers has caused work-related injuries in more than 78% of physical therapists and their assistants at rehabilitation facilities.^19^ Because of the work-related incidence of low back pain, 10% of female nurses reported having lost more than 1 month out of their total working days.^20^

The most common transfer method, which is known as a regular transfer or “getting-up transfer,” is defined as lifting the buttocks during transfer from one surface to another surface. Generally, the patient requires assistance at the point of lifting and rotating the buttocks during transfer. If interfering factors, such as a difference in the height of the surfaces, the width of the gap, or the presence of an obstacle, can be removed, the patient is more likely to be able to perform a transfer by scooting their buttocks laterally without lifting. This practice is referred to as the “lateral transfer.” We hypothesized that patients with hemiparetic stroke can improve their transfer independence by performing a lateral transfer under an environmental setup in which the transfer surfaces are at the same height with no gap, and with no obstacle between the surfaces. However, it is unclear whether there is a difference in the difficulty between the getting-up method and the lateral transfer method. If our hypothesis is correct, the lateral transfer approach would facilitate the independence of transfers and decrease the need for assistance during transfers. The aim of the present pilot study was to test our hypothesis among patients with hemiparetic stroke.

## Methods

### Participants

The current study used a cross-sectional design. Because this was a pilot study, the sample size was determined as the minimum number of participants based on previous reports that suggested recommendations for a sample size for pilot studies.^21,22^ Thirteen patients with hemiparetic stroke participated in the study (*M*_age_=64.8 years, *SD*=10.5, range: 47–79 years; Table 1). All patients suffered from hemiparesis following a cerebrovascular incident, and patients’ mean height and weight were 162.6 (*SD*=8.8 cm) and 57.4 (*SD*=11.6 kg), respectively. The exclusion criteria were as follows: presence of bilateral, cerebellar, and/or brain-stem lesions; severe upper-extremity pain or injury; severe injury of the lower extremity on the non-paretic side; other neuromuscular disease; and pressure sores.

### Getting-up transfer

**Figure 1:**
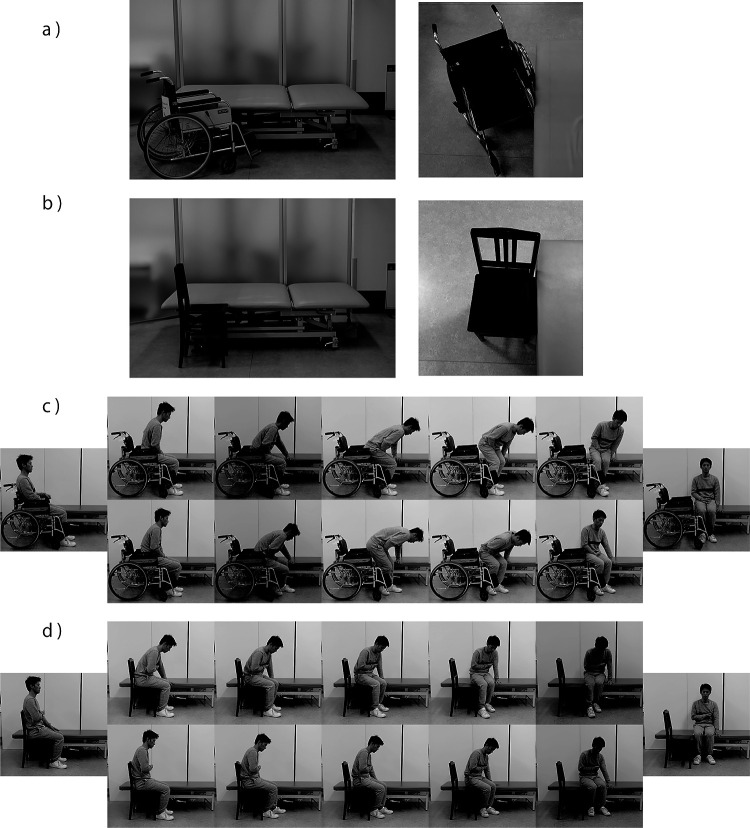
Experimental setup and procedure. Experimental setup for (a) wheelchair condition for getting-up transfer and (b) flat chair condition for lateral transfer, and procedure for (c) getting-up transfer and (d) lateral transfer. The leftmost panels of the figure show the starting position of transfer from the wheelchair/flat chair to the bed. The upper sequential pictures show the transfer procedure from the wheelchair/flat chair to the bed. The rightmost panels of the figure show the starting position of transfer from the bed to the wheelchair/flat chair. The lower sequential pictures show the transfer procedure from the bed to the wheelchair/flat chair. The model simulates a patient with right hemiparesis.

In the start position, the patient sat on the platform table with their feet on the floor. The patients were asked to transfer to/from the wheelchair in their usual way. The procedure for transferring from the platform table to the wheelchair was as follows (Figure 1c): (1) scoot forward to the edge of the platform table from the start position, (2) slide their buttocks laterally on the platform table closer to the wheelchair, (3) lean their trunk forward and lift their buttocks from the platform table, (4) rotate their buttocks to the direction of the wheelchair so that their head moves in the opposite direction to their buttocks, and (5) slowly sit on the wheelchair. In contrast, transfer from the wheelchair to the platform table was performed as follows: (1) grasp the armrest of the wheelchair with the non-paralyzed upper limb, (2) scoot forward to the edge of the wheelchair seat, (3) slide their buttocks within the wheelchair seat to be closer to the platform table, (4) lean their trunk forward and put their non-paralyzed hand on the platform table, (5) lift their buttocks from the wheelchair, (6) rotate their buttocks to the direction of the platform table, and (7) sit down slowly on the platform table.

### Lateral transfer

The starting position involved the patient sitting on the platform table with their feet on the floor. The patients were asked to transfer to/from the flat chair. The procedure was as follows (Figure 1d): (1) scoot forward to the edge of the platform table from the starting position, and (2) push on the platform table using their non-paretic upper limb to scoot their buttocks laterally to the surface of the flat chair. During the scooting movement, patients leaned their trunk slightly forward to move the weight of their buttocks to their feet. (3) Repeat the scooting motion several times until they are sitting on the flat chair. Conversely, the transfer from the flat chair to the platform table was performed as follows: (1) put their non-paretic upper limb on the platform table, and (2) scoot laterally from the starting position to the platform table repeatedly until they are sitting on the platform table.

### Experimental setup and procedure

A platform table, a standard wheelchair without a seat cushion, and a square flat chair (PI5; Yamaha Co., Ltd., Hamamatsu, Japan) with no armrests were used to perform the experiment. The height of the platform table and flat chair were adjusted to the same level as the height of the wheelchair seat. In the experiment using the wheelchair, it was positioned close to the platform table at 20–45° with the non-paretic side of the participant facing the platform table (Figure 1a). Before performing a transfer, both wheels of the wheelchair were locked in place. In the experiment using the flat chair, the chair was positioned parallel and very close to the platform table without a gap between the table and chair seat (Figure 1b). The chair was also positioned so that the paretic side of the patient faced the chair when sitting on the side of the platform table.

The participants performed the following four transfers: (1) transfer from the platform table to the standard wheelchair, (2) transfer from the standard wheelchair to the platform table, (3) transfer from the platform table to the flat chair, and (4) transfer from the flat chair to the platform table. Each transfer was performed according to the procedures described in the following section. One transfer was performed under each transfer condition. The patients were allowed to rest for a few minutes between trials according to their fatigue. One therapist closely supervised the patients to prevent a fall during transfers. According to the patient’s transfer ability, the therapist supported the lifting and/or rotating of the participant’s buttocks with minimum assistance. The transfers were video-recorded with a video camera for the assessment.

### Assessment

The Functional Independence Measure (FIM) transfer score was used as a measure of the primary outcome. The FIM transfer score provides a measure of the amount of physical assistance needed and level of independence observed during transfers based on a 1–7 ordinal scale and it has been shown to have high inter-rater reliability and validity.^23–26^ A score of 7 indicates “complete independence,” 6 indicates “modified independence,” 5 indicates “supervision,” 4 indicates “minimal assistance (subject 75%+),” 3 indicates “moderate assistance (subject 50%+),” 2 indicates “maximal assistance (subject 25%+),” and 1 equals “total assistance (subject 0%+).” Transfer ability was evaluated by three occupational therapists (including the therapist who supervised the transfer) using video footage of the transfer motions. In cases of disagreement between the raters, they discussed the score until a consensus was obtained. To examine the relationship between the transfer performance and the degree of motor impairment, the Stroke Impairment Assessment Set (SIAS) for motor function was used to assess motor impairment.^27,28^ The SIAS is a standardized measure of stroke impairment that consists of 22 subcategories, such as motor function, muscle tone, sensation, and pain. For motor function, each item is rated from 0 (*severely impaired*) to 5 (*normal*).

### Ethics

The study protocol was approved by the Ethics Review committee of Fujita Health University. All patients provided written informed consent before their participation in the study. This study was performed in accordance with the Declaration of Helsinki.

### Data analysis and statistical analysis

To analyze the SIAS finger score, 1a, 1b, and 1c of the SIAS finger score were transformed to 1, 2, and 3, respectively, while 2, 3, 4, and 5 of the SIAS finger score were transformed to 4, 5, 6, and 7, respectively, according to a previous study.^29^ To compare the FIM scores for getting-up transfer and lateral transfer, we used a Wilcoxon signed rank test. The relationship between the total SIAS motor function score and FIM transfer score was shown as a scatter diagram with a probability ellipse to analyze the trends between the motor impairment level and difficulty level for the two transfer types. All statistical analyses were performed with R (3.4.3; Institute of Statistical Mathematics, Tokyo, Japan). The statistical significance level was set at a *p*-value of .05.

## Results

**Figure 2:**
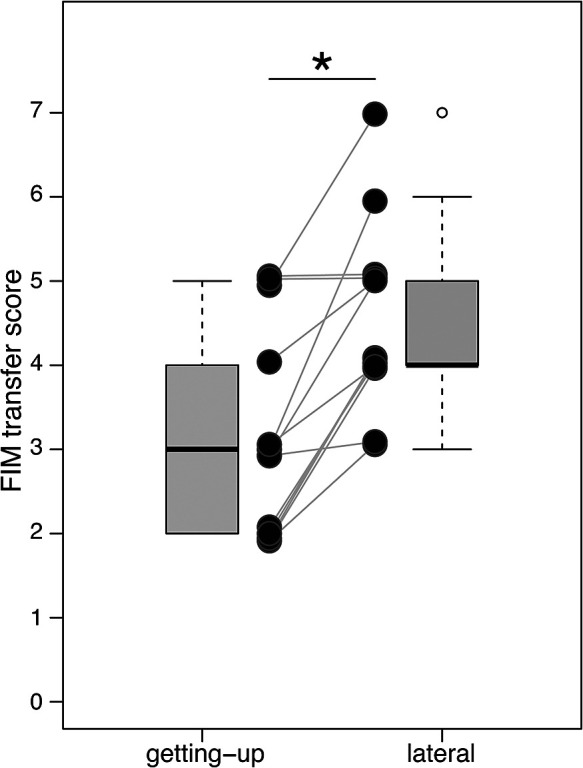
Functional Independence Measure (FIM) transfer scores for the getting-up and lateral transfer methods. The figure shows the difference in the FIM transfer score of each subject (line plot) and all subjects (boxplot). The horizontal axis indicates the transfer type and the vertical axis indicates the FIM transfer score. The center lines of the boxplot represent medians; box limits are the inter-quartile range from 25% and 75%. The boxplot whiskers extend 1.5 times the interquartile range from the first and third quartiles. Open circle (〇) and asterisk (*) in the boxplot represent outlier and p<0.05, respectively.

**Figure 3:**
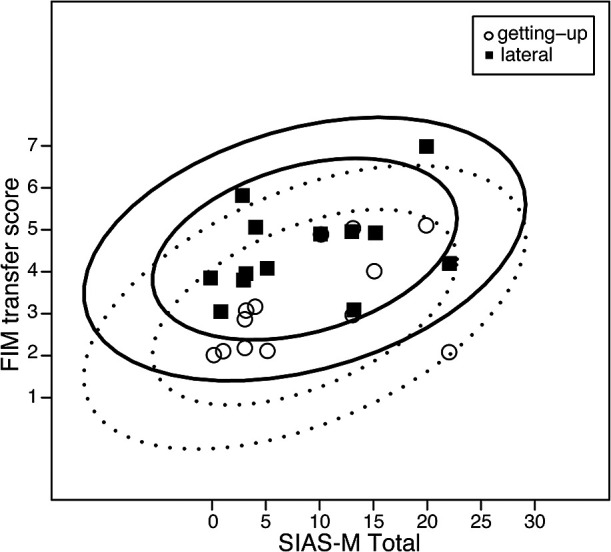
Scatter diagram with probability ellipses for stroke impairment. Scatter diagram for the Stroke Impairment Assessment Set motor function total (SIAS-M Total) score and Functional Independence Measure (FIM) transfer score for the getting-up transfer (〇) and lateral transfer (■) methods. The dotted lines show the 95% and 80% probability ellipses for the getting-up transfer method. The solid lines show the 95% and 80% probability ellipses for the lateral transfer method.

All patients performed all transfers without any adverse events. The FIM transfer scores for the getting-up and lateral transfer methods are shown in Figure 2. The FIM transfer score was higher for the lateral transfer compared with the getting-up transfer in almost all patients. The median FIM score for the getting-up transfer was 3 (range 2–5). For the lateral transfer, the median FIM score was 4 (range 3–7). Statistical analysis revealed that the FIM score was significantly higher in the lateral transfer method than in the getting-up transfer method (*p*=.002). The relationship between the FIM transfer score for each method and the total SIAS motor function score is shown in Figure 3. The probability ellipse shifted in the upward direction for the lateral transfer compared with the getting-up transfer, indicating that the lateral transfer improved the degree of independence and reduced the degree of assistance, even in participants with a similar degree of lower-limb motor paralysis.

## Discussion

One of the most important aspects of rehabilitation practice is ensuring safety and independence in activities of daily living. The aim of the present study was to test whether the level of independence in performing a transfer differs between two methods for a bed-to/from-wheelchair transfer in patients with hemiparetic stroke. The FIM transfer scores were significantly higher for the lateral transfer than for the getting-up transfer. These results suggested that the lateral transfer was safe for use in transferring patients with stroke. Moreover, the results detailed how much lateral transfer contributed to the improvement of independence in transferring, which is clinically useful in deciding the type of vehicle to use, based on patients’ ability.

In the commonly used getting-up transfer method, to perform a transfer from a wheelchair to another surface, patients must lift their buttocks to avoid hitting the armrest and wheel of the wheelchair^5,6,30–34^. Conversely, the lateral transfer method requires patients to raise their center of gravity only a minimal amount and allows them to transfer to another surface by moving their center of gravity mainly in a lateral direction on the level surface. A particularly important difference may be that the lateral transfer method does not require high motor functioning of the lower limbs. Previous studies reported that the lower limbs play an essential role in supporting body mass and controlling sitting balance during major forward movements of the center of gravity in a sitting position that occur during getting-up transfers.^35,36^

The lateral transfer method may also reduce the risk of transfer-related pain and accidents such as falls in patients with hemiparetic stroke. When wheelchair users with poor trunk and lower-limb function transfer from a seat to another surface, they must rely on their upper extremities for stability and mobility. Their soft tissue structures are exposed to overuse during transfers because the shoulder becomes a weight-bearing joint.^7^ The overuse of an upper limb can contribute to the pathogenesis of joint pain.^7^ Regarding transfer-related falls among inpatients in a rehabilitation center, Saverino et al. reported that falls occur most frequently during standing up without assistance.^37^ As a lateral transfer is achieved by a narrow range of movements in the antero-posterior and vertical directions, this might reduce the burden and subsequent pain of the upper limbs and the risk of falling compared with the getting-up transfer method.

In addition, the present results suggest that the requirement for transfer assistance was decreased by using the lateral transfer method. As described above, work-related injuries to healthcare workers during transfers are prevalent in healthcare,^14–17^ with low back pain being the most common result.^38^ This injury typically occurs when the buttocks of the care receiver are lifted during a transfer.^38^ Thus, the present findings may be useful for the prevention of work-related injuries associated with transfer assistance.

A major clinical implication drawn from the present study is that the lateral transfer approach has the potential to improve the independence of transfers and decrease the need for assistance during transfers in various areas, such as the bed, toilet, and bathtub. A standard wheelchair has several barriers to this approach; in particular, the wheel, armrest, and height difference between the wheelchair and bed inhibit this approach. To overcome the difficulties involved in the getting-up transfer method when using a standard wheel chair, various assistive tools (e.g., transfer boards)^39,40^ and a wheelchair with a removable arm-rest type^41^ have been used. However, although these approaches can partially reduce the barriers, it cannot completely eliminate them. Considering the current findings, we argue for the development of novel transfer assist devices that eliminate such barriers completely and make the environment more conducive for lateral transfers. When a new mobility/transfer assistive device that can promote the lateral transfer method is proposed,^42^ rehabilitation workers could begin to recommend the use of the device based on the evidence presented in this study.

The results of the current study revealed that the lateral transfer was easier than the getting-up transfer in patients with hemiparetic stroke, even in participants with a similar degree of lower-limb motor paralysis. However, the present pilot study involved several limitations that should be considered. Because of the small sample size, it was not possible to delineate those characteristics of wheelchair users that are most beneficial for the lateral transfer method. Further research with a larger number of participants with various types and levels of impairment is needed to investigate which characteristics inhibit and facilitate the use of the lateral transfer method. Moreover, in future research, a multicenter randomized controlled trial should be carried out with an appropriately powerful sample size, given that this pilot study has shown that lateral transfer might be effective. Conducting further research with an appropriate sample size, in addition to a wider range of severities and disorders, would provide further clinical evidence of the advantages of the lateral transfer method.

## Figures and Tables

**Table1:** Participants’ characteristics (N=13)

ID	Gender	Age (years)	Height (cm)	Weight (kg)	TO (days)	Diagnosis	Paretic side	SIAS					Cognitive impairments	MMSE
								HF	KE	FT	KM	FF		
1	Male	68	164	58.2	51	CH	Right	4	4	3	3	4	MD	27
2	Female	69	156	47.6	21	CH	Left	0	0	0	0	0	USN, AD, MD, ED	20
3	Female	47	167	58.3	23	CH	Left	1	2	0	0	0	None	29
4	Male	74	160	49.6	40	CI	Left	1	1	1	1	0	AD, USN	18
5	Male	66	165	50.5	13	CH	Left	1	0	0	0	0	AD, USN	26
6	Male	56	165	53.3	30	SAH	Left	1	1	0	3	3	MD, AD, ED	25
7	Male	68	170	55.4	27	CH	Right	2	2	0	1	0	AD, Asomatognosia	23
8	Female	67	161	45.2	117	CI	Right	3	2	2	2	2	Motor aphasia, AD, MD, USN	18
9	Male	76	156	47	45	CH	Right	1	1	0	1	0	Motor aphasia, USN, AD	unmeasurable
10	Female	54	150	64.4	152	CH	Left	1	1	0	1	0	AD	30
11	Female	79	153	55.3	37	CH	Right	4	4	4	4	4	None	26
12	Male	71	162	78.5	36	CI	Left	2	3	3	2	1c	AD	27
13	Male	47	185	82.6	104	CH	Left	3	3	3	2	2	Motor aphasia	20
Mean		64.8	162.6	57.4	53.5									24.1
(*SD*)		(10.5)	(8.8)	(11.6)	(42.8)									(4.2)

*Note*. TO, Time since stroke onset; CI, Cerebral infarction; CH, Cerebral hemorrhage; SAH, Subarachnoid hemorrhage; SIAS, Stroke Impairment Assessment Set; HF, Hip flexion test; KE, Knee extension test; FT, Foot tap test; KM, Knee mouth test; FF, Finger function test; MD, Memory disorder; USN, Unilateral spatial neglect; AD, Attentional disorder; ED, Executive dysfunction; MMSE, Mini-Mental State.

## Funding Statement

The authors received no support for this work.
